# Supplementary material for: The effect of social support on academic performance among adolescents: The chain mediating roles of self-efficacy and learning engagement
Source: PLoS One. 2024 Dec 31;19(12):e0311597. doi: 10.1371/journal.pone.0311597 (PMC11687781; doi:10.1371/journal.pone.0311597)
Supplement: Attachment — Submitted filename: Respondse to reviewers.doc [file pone.0311597.s002.doc]

**Response to Reviewers**

Title: The Chain Mediating Roles of Self-efficacy and Learning Engagement

Authors: Xiangping Zhang, Wensheng Qian

Manuscript ID: PONE-D-24-10692

**Dear reviewers,**

Thank you very much for your comments and professional advice. We agree with the reviewers' suggestions and will incorporate the recommended changes into the manuscript. We believe that these revisions will significantly strengthen our manuscript and provide a more robust foundation for our claims. We hope that our work can be improved again. Furthermore, we would like to show the details as follows:

**Reviewer 1#**

**Abstract Section**

**Comments:** In the abstract section, authors need to improve the wording, break down sentences, simplify sentence structure, switch tenses, etc. to enhance accuracy, logic, and readability.

**Response:** Thanks for your suggestions, you have provided us with very valuable advice to improve the quality of the abstract section. We have used your comments to improve its accuracy, logic, and readability.

**1.** Background was revised as “Although the effects of social support, self-efficacy, and learning engagement on academic performance are well-documented, there is still limited understanding of the specific mechanisms through which social support influences academic performance through self-efficacy and learning engagement.”

**2.** Purpose was revised as “This study aims to investigate the relationship between social support and adolescents' academic performance based on the Social Cognitive Theory.”

**3.** The method was revised as “The data was collected from 265 participants (mean age=13.47 years, SD = 0.5) in four middle schools, in the Shandong Province of China in June 2023.”

**4.** Conclusion was revised as “To enhance adolescents’ academic success, appropriate interventions should be implemented for teachers, parents, or other social factors, to construct a positive learning environment in and out of school to improve adolescents’ self-efficacy and learning engagement.”

**Introduction Section**

**Comments:** **In the Introduction section, authors need to rephrase, combine, streamline, remove redundancy, and tighten, clarify, and simplify the sentences.**

**1.** Rephrase “In China, test-based education is one of the most important methods of selecting talent, which puts many students under a lot of academic pressure [1].“

**2.** Combine and streamline: "During this special academic climate, middle school students are facing challenges such as how to form learning habits and behaviors, deal with obstacles in study, and reduce learning pressure.”

**3.** Remove redundancy and tighten: "It is evaluated and measured using a variety of criteria to determine students’ academic performance and progress, including grade assessment, academic skills and abilities, and academic outcomes [4, 5, 6].”

**4.** Clarify: "To fill this gap, our study aims to comprehensively check the interplay between social support, self-efficacy, learning engagement, and academic performance.”

**5.** Simplify: "The study’s goal is to give a comprehensive knowledge of the cumulative influence of social support, self-efficacy, and learning engagement on academic success.”

**6.** Simplify: "The Social Cognitive Theory (SCT) gives a theoretical structure for developing a chain mediation model in this study.”

**Response:** Thanks for your corrections, it was revised as:

**1.** In China, middle schools are committed to providing quality education as an essential component of compulsory education, and as such they pay special attention to the development of intellectual education. For this reason, their assessment methods focus primarily on examinations for assessing student progress, learning, and selecting talent [1, 2], which does emphasize the importance of examination results in the education process.

**2.** However, it cannot be ignored that this evaluation method also brings challenges to middle school students in forming learning habits, overcoming study obstacles, and reducing learning pressure [3].

**3.** It is measured through various criteria, including grade assessments, academic skills, and outcomes [13, 14].

**4.** To fill this gap, our research will look at the relationship between social support, self-efficacy, learning engagement, and academic performance, as well as the role of self-efficacy and learning engagement as mediators.

**5.** The study aims to provide a comprehensive understanding of the combined influence of social support, self-efficacy, and learning engagement on academic success with SCT.

**6.** The Social Cognitive Theory (SCT) proposed by Bandura in 1986, provides the theoretical framework for developing a chain mediation model in this study.

**Literature Section**

**Comments:** In the literature section, authors need to add some latest ref.

**Response:** As suggested by the reviewer, we have added more references to support the ideas proposed.

**Method Section**

**Comments 1:** **In the Method section, authors need a citation for the recommended sample size standards in structural equation modeling (SEM) to support the claim of stability and reliability of the model estimate.**

**Response 1:** We thank the reviewer for this helpful recommendation. We added the sample size selection criteria on Page 10 Lines 179-180:” It is explained that the proper sample size should be at least ten times the total number of variables observed due to the Structure Equation Modeling (SEM) recommendation [57].”

**Comments 2:** **The sentence "The selection of these schools was based on their willingness to participate and their representation of diverse socioeconomic backgrounds." should specify how diversity in socioeconomic backgrounds was assessed and measured.**

**Response 2:** Thank you for your question. The four middle schools chosen in our research were located in the urban and rural areas of Shandong Province, China. First of all, the geographical distribution of the four junior secondary schools itself reflects the diversity of China's socioeconomic structure. Secondly, factors such as the composition of students and the quality of education of four middle schools also reflect the diversity of China's socioeconomic structure. Furthermore, the diversity in socioeconomic backgrounds of the participating middle schools was measured by the place of residence of students, parental education level, and parental monthly income. In the text, we added the explanation on Page 10 Lines 185-188:” Meanwhile, these middle schools are representative of diverse socioeconomic backgrounds including a mix of urban and rural locations, varied student populations, and diverse academic performance levels, which ensure a balanced representation of both genders and different grade levels within the specified age range.”

**Comments 3:** **Authors should mention the time frame during which the data collection took place.**

**Response 3:** Thanks for your information. These participants were recruited from June 1 to June 30, 2023. we added it on Page 8 Lines 169-170.”

**Comments 4:** **Add a section detailing the statistical methods and software used for data analysis.**

**Response 4:** Thank you for your question. we added the explanation on Pages 12-13 Lines 227-233:” Data were analyzed using Amos 24.0 and SPSS 24.0. First, the Harman single-factor test was used to assess the possibility of common method bias. The sample’s characteristics were then appropriately reflected using descriptive analysis. Following that, a structural equation modeling (SEM) analysis was run to assess both the measurement and structural models. The measurement model was validated using confirmatory factor analysis, while the structural model was investigated using goodness-of-fit indices and path coefficients. Finally, the significance of mediating effects was determined using the bootstrapping method.”

**Comments 5:** **Describe how missing data was handled and any assumptions made during the analysis.**

**Response 5:** Thanks for your information. we added the explanation on Page 10 Lines 179-183:” We distributed 300 survey forms, but only 265 (92.6%) completed survey forms were received for further analysis. 14 (4.7%) were rejected because of incomplete records and missing answers.”

**Conclusion Section**

**Comments:** **Mention the conclusion in one paragraph.**

**Response:** We are so grateful for this suggestion. As suggested by the reviewer, we have revised the conclusion in one paragraph as follows.

**Conclusion**

The study set out to find the relationship between social support and academic performance in the context of China's test-based culture. The current data suggest that adolescents' academic performance is positively and directly impacted by social support, meanwhile, learning engagement and self-efficacy play the role of chain mediators in the association between social support and academic performance. Also, the relationship and effect of various variables are further described with the help of SCT. As evidenced by previous research studies, this article is unique and provides new insights regarding the role of social support, self-efficacy, learning engagement, and academic performance in the special academic climate of China. This research contributes to the existing academic performance literature by confirming a mediating process in the relationship among academic performance, social support, self-efficacy, and learning engagement. In the test-oriented learning climate of China, middle school students who get more help from parents, teachers, or peers can easily achieve self-efficacy and engage in learning, which in return achieve better academic performance in learning. In addition, the result of the research enhances the understanding of the relationship between social support and academic performance based on the SCT, which emphasizes the significance of social elements in determining students' self-efficacy and involvement in the learning process. Regarding the practical implications, it is conducive for educational practitioners to easier comprehend the measures of improving students' academic performance from the angle of social factors such as educators, parents, peers, etc. Regarding social support, teachers should foster an inclusive and internal classroom environment to promote respect, empathy, and cooperation in learning, such as carrying out peer mentoring programs, and cooperative learning activities. Parents should create a supportive learning environment at home, for example, building a focused atmosphere, providing dedicated study space, and minimizing external distractions. In regards to self-efficacy, teachers or parents should encourage students to participate in problem-solving activities that connect everyday learning to real-life experiences, as well as motivate adolescents to take on challenges and solve problems [80]. In addition, teachers or parents should provide timely and constructive feedback that allows students to monitor their learning progress and adjust their strategies accordingly, thereby raising students' self-efficacy. Concerning learning engagement, teachers, parents, and other social factors should work together to gain a better understanding of adolescents' needs and use tactics and skills to increase their engagement in learning through meaningful practical activities.

**Reviewer 2#**

**Introduction Section**

**Comments 1:** **Improve the coherence between each paragraph of the Introduction.**

**Responsev1:** We are very sorry for this kind of mistake and we have carefully reviewed the Introduction Section and the entire manuscript to improve the coherence between each paragraph.

**Comments 2:** **State the gaps in the study.**

**Response 2:** We are sorry to bother you with our negligence and we have added the statement of gaps in the study on Pages 4-5 Lines 63-69:” However, there is still a lack of understanding about the specific mechanisms by which social support, self-efficacy, learning engagement, and academic performance interact with one another, particularly the mediation effect of self-efficacy and learning engagement on social support and academic performance in adolescents. To fill this gap, our research will look at the relationship between social support, self-efficacy, learning engagement, and academic performance, as well as the role of self-efficacy and learning engagement as mediators.”

**Comments 3: Make the literature review section a separate paragraph.**

**Response 3:** We appreciate the reviewer for this recommendation and we have made the literature review section a separate paragraph on Pages 5-9, Line 88-160.

**LITERATURE REVIEW**

**Social support and academic performance**

Relevant research has shown that social support is a significant predictor of academic performance. It has been found that social support can significantly improve people's self-confidence [26], and motivation [27]. Researchers also have proved that parental involvement and encouragement can enhance students' focus and motivation, leading to improved academic outcomes [28, 29]. Additionally, a supportive family environment provides stability and emotional support, helping students obtain higher academic grades [30]. Social support from peers also plays a crucial role in influencing academic performance [31, 32]. For example, Wentzel noted that interactions with peers who demonstrate positive learning attitudes and behaviors can stimulate students' motivation and enhance their academic performance [33]. Importantly, it has been revealed that social support from educators has a significant positive impact on academic performance [34]. Research has indicated that individualized tutoring and additional assistance provided by teachers can address students' specific learning needs and provide effective learning guidance, thereby promoting academic progress [35]. Overall, these findings emphasize the critical role of social support in academic performance, highlighting that adolescents who get support from their parents, peers, and teachers are more inclined to achieve success in their academic pursuits. On this basis, we propose the following hypothesis.

**Comments 4:** **The lines 66~98 need to be greatly condensed and summarised in no more than one paragraph.**

**Response 4:** Thanks for your suggestions, we have used your comments to improve its accuracy, logic, and readability on Page 5 Lines 70-87:

“The Social Cognitive Theory (SCT), proposed by Bandura in 1986, provides the theoretical framework for developing a chain mediation model in this study. According to this theory, human behavior is influenced by three variables: personal factors (e.g., self-efficacy), behavioral factors (e.g., learning engagement and achievement), and environmental factors (e.g., social support ). Namely, individual behavior is driven and moderated by personal, behavioral, and environmental elements [23]. Past investigations have used SCT to probe into how personal cognitive or environmental factors affect academic performance among adolescents [24, 25], with less attention paid to the interplay between social support, self-efficacy, learning engagement, and academic performance within the framework of SCT. Based on the above, the study aims to provide a comprehensive understanding of the combined influence of these four factors with SCT.

This study takes a broader approach, examining the interrelationships and mediating effects of social support, self-efficacy, learning engagement, and academic performance. As a result, this study differs from previous research, which focused on the impact of single factors on academic performance. The significance of this study stems from its contribution to bridging the existing research gap and providing a better understanding of the factors that influence students' academic success. By investigating the interactive effects and mediating roles of these factors, the study proposes to offer valuable insights into the complex dynamics that influence academic performance among adolescents.”

**Method Section**

**Comments 1:** **Please add sampling techniques.**

**Response 1:** We appreciate the reviewer for this kind of recommendation and we have added the sampling techniques on Page 9-10 Line 168-178:” The present study employed the simple random sampling method to recruit Chinese middle school students to voluntarily take part in the questionnaire collection. The data was collected from 1 June to 30 June 2023. Firstly, a structured questionnaire was administered to the students during regular school hours in a classroom setting. The researchers provided clear instructions, addressing any queries or concerns raised by the participants. The students were encouraged to respond honestly, with the assurance of confidentiality regarding their responses. Secondly, the study included the collection of students' academic performance data. The academic performance was measured using scores from the final exams in Chinese, Math, and English subjects. To ensure comparability and facilitate analysis across different subjects, the overall scores, ranging from 0 to 120, were standardized. Then standardized scores were utilized as observed variables to measure academic achievement in the study.”

**Comments 2: Participant characteristics should be stated in the methodology.**

**Response 2:** Thank you for your suggestion and we have added the statement of the participants’ characteristics on Pages 10-11, Lines 189-204.:

**“Sample characteristics**

The sample consisted of 265 participants selected from four middle schools in eastern China. The average age of the participants was 13.71 years (SD=0.5, range=13-14 years). As shown in Table 2, there were 128 female students (48.3%) and 137 male students (51.7%). There were 93 students in the first year (35.1%), 91 in the second year (34.3%), and 81 in the third year (30.6%). In terms of residence, 137 families (51.7%) lived in urban areas, while 128 families (48.3%) lived in rural areas. Concerning parental education, 159 fathers (60%), and 192 moms (72.5%), had completed junior high school or less. 77 fathers (29.1%) and 51 mothers (19.2%) had received education at the high school or technical school level. Additionally, 17 fathers (6.4%) and 12 mothers (4.5%) had received education at the college level. Furthermore, 12 fathers (4.5%) and 10 mothers (3.8%) had received education at the undergraduate level or above. In terms of monthly income, 52 families (19.6%) had an income below 3000 yuan, 124 families (46.8%) had an income between 3000 and 5000 yuan, 68 families (25.7%) had an income between 5000 and 10000 yuan, and 21 families (7.9%) had an income above 10000 yuan. Overall, the sample in this study is representative of the target population since it closely matches the demographic features and distribution seen in the region.”

**Comments 3:** **APA formatting should not be included in the text.**

**Response 3:** We are very sorry for our incorrect formatting and it has been rectified.

**Discussion Section**

**Comments 1:** **Suggest** **additional subheadings for research discussions.**

**Response 1:** We thank the reviewer for this helpful recommendation and we have added the additional subheadings for research discussions on Pages 17-20, Lines 314-375:” **DISCUSSION**

According to SCT, the present study looked into the link between social support, self-efficacy, learning engagement, and academic performance among adolescents. To understand the mechanism through which social support affects academic performance, the study sought to investigate the potential mediating effects of self-efficacy and learning engagement in the relationship between social support and academic performance in the exam-oriented education setting. The study's findings are presented below.

**Direct Effects Analysis…**

**Indirect Effects Analysis…**

**Chain mediator effect...”**

**Comments 2:** **Please add a section on "Conclusion".**

**Response 2:** We appreciate the reviewer for this kind of recommendation, and have added a section on “Conclusion” on Pages 20-21, Lines 376-406:

**“CONCLUSION**

The study set out to find the relationship between social support and academic performance in the context of China's test-based culture. The current data suggest that adolescents' academic performance is positively and directly impacted by social support, meanwhile, learning engagement and self-efficacy play the role of chain mediators in the association between social support and academic performance. Also, the relationship and effect of various variables are further described with the help of SCT. As evidenced by previous research studies, this article is unique and provides new insights regarding the role of social support, self-efficacy, learning engagement, and academic performance in the special academic climate of China. This research contributes to the existing academic performance literature by confirming a mediating process in the relationship among academic performance, social support, self-efficacy, and learning engagement. In the test-oriented learning climate of China, middle school students who get more help from parents, teachers, or peers can easily achieve self-efficacy and engage in learning, which in return achieve better academic performance in learning. In addition, the result of the research enhances the understanding of the relationship between social support and academic performance based on the SCT, which emphasizes the significance of social elements in determining students' self-efficacy and involvement in the learning process. Regarding the practical implications, it is conducive for educational practitioners to easier comprehend the measures of improving students' academic performance from the angle of social factors such as educators, parents, peers, etc. Regarding social support, teachers should foster an inclusive and internal classroom environment to promote respect, empathy, and cooperation in learning, such as carrying out peer mentoring programs, and cooperative learning activities. Parents should create a supportive learning environment at home, for example, building a focused atmosphere, providing dedicated study space, and minimizing external distractions. In regards to self-efficacy, teachers or parents should encourage students to participate in problem-solving activities that connect everyday learning to real-life experiences, as well as motivate adolescents to take on challenges and solve problems [80]. In addition, teachers or parents should provide timely and constructive feedback that allows students to monitor their learning progress and adjust their strategies accordingly, thereby raising students' self-efficacy. Concerning learning engagement, teachers, parents, and other social factors should work together to gain a better understanding of adolescents' needs and use tactics and skills to increase their engagement in learning through meaningful practical activities.”

**Literature Section**

**Comments:** **In the literature section, authors need to add some latest ref.**

**Response:** As suggested by the reviewer, we have added more references relevant to the study.

We sincerely appreciate the time and effort invested by the reviewers in evaluating our manuscript. We look forward to any additional feedback or suggestions.
