# Supplementary material for: The effect of social support on academic performance among adolescents: The chain mediating roles of self-efficacy and learning engagement
Source: PLoS One. 2024 Dec 31;19(12):e0311597. doi: 10.1371/journal.pone.0311597 (PMC11687781; doi:10.1371/journal.pone.0311597)
Supplement: Attachment — Submitted filename: Response to Reviewers.docx [file pone.0311597.s003.docx]

**Response to Reviewers**

**Title:** The effect of social support on academic performance among adolescents: The chain mediating roles of self-efficacy and learning engagement

**Authors:** Xiangping Zhang, Wensheng Qian

**Manuscript ID:** PONE-D-24-10692

**Dear reviewer,**

Thank you for your letter and for reviewer’ comments concerning our manuscript entitled “The effect of social support on academic performance among adolescents: The chain mediating roles of self-efficacy and learning engagement”. All of those remarks are relevant and beneficial for the purpose of rewriting and enhancing our paper. We have thoroughly examined every comments and have made diligent corrections. We are confident that these adjustments will greatly enhance our manuscript and establish a strong basis for our assertions. We aspire for continued enhancement of our work.The responses to the reviewer's comments are as follows:

**Reviewer 1#**

**Abstract Section**

**Comments:** Authors must adhere to the prescribed structure for the abstract, which includes sections on the purpose, method, results, findings, practical implications, and originality/value.

**Response:** We appreciate your ideas, since they have given us great guidance on enhancing the quality of the abstract part. We have made revisions to this section as follows:

**Purpose**- While the impact of social support on academic performance is acknowledged, the specific mechanisms by which social support affects academic performance, particularly through self-efficacy and learning engagement, remain poorly understood. This study aims to examine the correlation between social support and academic achievement among Chinese middle school students, framed within the Social Cognitive Theory. It also seeks to explore the mediating roles of self-efficacy and learning engagement in this relationship.

**Method**- Data was collected from 265 individuals (mean age = 13.47 years, SD = 0.5) in four middle schools in Shandong Province, China in June 2023, using the simple random sample technique. Participants completed the questionnaires independently, and the data was analyzed using the structural equation model (SEM) in AMOS 24.0 and SPSS 24.0.

**Results**- Social support and academic performance have a direct and significant relationship with the SCT among middle school students. In addition, social support indirectly and positively affects academic performance through self-efficacy and learning engagement. The results also highlight self-efficacy as a key factor linking social support with academic performance.

**Practical implications**- This study offers valuable insights into the role of social support in Chinese middle school students’ academic achievement, particularly by examining the impact of self-efficacy and learning engagement. These valuable findings may guide policymakers in creating a supportive educational environment both inside and outside the classroom, enhancing adolescents’ self-confidence and engagement in learning.

**Originality**- This study contributes to the theoretical understanding of social support by investigating the mechanisms through which it impacts academic achievement. It clarifies the complex interactions among social support, self-efficacy, learning engagement, and academic achievement, with particular emphasis on the mediating roles of self-efficacy and learning engagement within the Chinese context.

**Introduction Section**

**Comments:** Revise the Introduction section to improve linguistic conciseness, streamline the information, and ensure clarity.Introduction: lines 20-21 the sentence is clear but could be made more concise. Lines 24-26 The phrase "it cannot be ignored that" can be simplified. Lines 27-29 Consider rephrasing for better flow. Lines 29-30 the sentence is lengthy and could be broken into two for clarity. Lines 34-36 rephrase for clarity and remove redundancy. Lines 45-47 Clarify the importance of self-efficacy and learning engagement. Lines 54-59 Combine and streamline for clarity. lines 63-66 Streamline and emphasize the research gap.

**Response:** Thanks for your corrections, it was revised as:

**Lines 20-21 are revised as:** Within China's compulsory education system, middle school serves as a crucial intermediary, bridging different levels of education. At this stage, academic performance is a significant indicator of students' information acquisition and their potential for future studies. It is used to evaluate student progress, learning, and talent selection [1, 2].

**Lines 24-26 are revised as:** In China, academic success is commonly assessed through exam scores in subjects such as Chinese, Math, and English [3, 4]. Nevertheless, this assessment approach presents challenges for middle school students in developing study routines, overcoming academic difficulties, and managing time and anxiety [5]**.**

**Lines 27-29 are revised as:** Learning is predominantly a cognitive endeavor that involves the acquisition of knowledge through various social activities [6]**.** Chinese students, however, have limited opportunities for social engagement, primarily confined to interactions within the classroom, on campus, and within their families.

**Lines 29-30 are revised as:** Social support refers to the resources obtained through social interactions, which reflect the degree of connection between an individual and their community.

**Lines 34-36 are revised as:** It serves as a defensive shield against negative emotions and stress [7, 8]**,** providing a sense of being valued and supported by others when needed [9].

**Lines 45-47 are revised as:** These two psychological constructs are closely related to academic performance [20]. Self-efficacy acts a crucial role in motivating individuals to achieve their goals, encouraging them to take risks, and reaching their academic outcomes [21,22].

**Lines 54-59 are revised as:**Learning engagement refers to the active participation of students in the educational process, which positively impacts on academic performance. Engaged students are typically more motivated, committed, and willing to invest the effort needed to participate in discussions, solve problems, and achieve academic success [25, 26].

**Lines 63-66 are revised as:** However, little attention has been paid to the influence of social support on the academic performance of Chinese middle school students. Furthermore, this study identifies a gap in understanding the precise mechanisms by which social support affects academic performance through self-efficacy and learning engagement, as outlined in relevant theoretical frameworks.

**Literature Section**

**Comments:** In the literature section, authors need to add some latest ref.

**Response:** As suggested by the reviewer, we have added more references to support the ideas proposed.

2. Zeb A, Ullah R, Karim R. Exploring the role of ChatGPT in higher education: opportunities, challenges and ethical considerations. The International Journal of Information and Learning Technology. 2024; 41(1):99-111. https://doi.org/10.1108/ijilt-04-2023-0046
6. Rehman FU, Ismail H, Al Ghazali B M, Asad MM, Shahbaz MS, Zeb A. Knowledge management process, knowledge based innovation: Does academic researcher’s productivity mediate during the pandemic of covid-19?. Plos one. 2021;16(12):e0261573. https://doi.org/10.1371/journal.pone.0261573
22. Rehman FU, Zeb A. Investigating the nexus between authentic leadership, employees’ green creativity, and psychological environment: evidence from emerging economy. Environmental Science and Pollution Research. 2023; 30(49):107746-107758. https://doi.org/10.1007/s11356-023-29928-1
30. Zeb A, Gan GGG, Javaid M, Khan MN, Khan AU, Gul S. The interplay between supervisor support and job performance: Implications of social exchange and social learning theories. Journal of Applied Research in Higher Education. 2023; 15(2):429-448. https://doi.org/10.1108/jarhe-04-2021-0143
42. Zeb A, Gan GGG, Wei OJ, Karim R. Examining the nexus between situational factors and job performance through the mediating role of work engagement and self‐efficacy. Journal of Public Affairs. 2024; 24(2):e2915. <https://doi.org/10.1002/pa.2915>

**Results Section**

**Comments:**If the HTMT technique is applicable in AMOS, it might be mentioned to address the issue of discriminant validity.

**Response:**In response to the reviewer's suggestion, we have implemented the HTMT technique to resolve the problem of discriminant validity.

To validate discriminant validity, this study employed the Heterotrait-Monotrait (HTMT) criterion. According to Kline [68], and Henseler et al. [69], an acceptable HTMT value should remain below 0.85. **Table 4** indicates that discriminant validity was achieved.

**Table 4. Heterotrait-Monotrait Ration (HTMT)**

| **Potential variable** | **Social support** | **Self-efficacy** | **Learning engagement** |
| --- | --- | --- | --- |
| Social support |  |  |  |
| Self-efficacy | 0.403 |  |  |
| Learning engagement | 0.508 | 0.326 |  |

**Discussion Section**

**Comments:** Two headings, "Practical Implications" and "Theoretical Implications," can be included in Discussion section.

**Response:** We are so grateful for this suggestion. As suggested by the reviewer, we have revised the discussion as follows.

**Theoretical implication**

This study contributes to the existing theoretical knowledge by underscoring the influence of social support on the academic performance of middle school students within the framework of Social Cognitive Theory. It supports the notion that social support plays a significant role in academic achievement by elucidating the complex interactions among social support, self-efficacy, learning engagement, and academic performance. Furthermore, this research builds on previous empirical studies that have established a link between social support and academic performance. By confirming these findings within the context of the Chinese compulsory education system and emphasizing the mediating roles of self-efficacy and learning engagement, this study enhances the theoretical understanding of the relationship between social support and academic achievement among middle school students in China.

**Practical implication**

Regarding the practical implications, it is essential for educational practitioners to understand how to enhance students' academic achievement by considering social factors such as the roles of teachers, parents, and peers. To strengthen social support, teachers should create an inclusive and cohesive classroom environment that fosters respect, understanding, and collaboration among students. This can be accomplished through initiatives like peer mentorship programs and collaborative learning activities. Parents also play a vital role in establishing a conducive learning environment at home. They can do this by promoting a focused atmosphere, designating a dedicated study area, and minimizing external distractions. To enhance self-efficacy, it is important for both teachers and parents to encourage students to participate in problem-solving activities that relate to real-life situations. Additionally, they should motivate adolescents to embrace challenges and seek solutions, thereby helping them develop confidence in their abilities [86]. Furthermore, it is essential for educators and guardians to provide timely and constructive feedback that allows students to monitor their learning progress and adjust their approaches accordingly. This kind of feedback can significantly enhance students' self-efficacy and belief in their own abilities. In terms of learning engagement, it is important for teachers, parents, and other social variables to collaborate in order to develop a comprehensive understanding of adolescents' needs. By employing effective strategies and techniques, they can foster greater involvement in learning through meaningful and practical activities. This coordinated effort will not only engage students more deeply but also support their overall academic development.

We deeply value the time and effort dedicated by the reviewers in assessing our article. We eagerly anticipate any further feedback or recommendations.
